# Niacin alleviates TRAIL-mediated colon cancer cell death via autophagy flux activation

**DOI:** 10.18632/oncotarget.5374

**Published:** 2015-10-22

**Authors:** Sung-Wook Kim, Ju-Hee Lee, Ji-Hong Moon, Uddin M.D. Nazim, You-Jin Lee, Jae-Won Seol, Jin Hur, Seong-Kug Eo, John-Hwa Lee, Sang-Youel Park

**Affiliations:** ^1^ Biosafety Research Institute, Department of Biochemistry, College of Veterinary Medicine, Chonbuk National University, Jeonju, Jeonbuk 561–756, South Korea

**Keywords:** niacin, autophagy, TRAIL, death receptor, mitochondrial membrane potential

## Abstract

Niacin, also known as vitamin B_3_ or nicotinamide is a water-soluble vitamin that is present in black beans and rice among other foods. Niacin is well known as an inhibitor of metastasis in human breast carcinoma cells but the effect of niacin treatment on TRAIL-mediated apoptosis is unknown. Here, we show that niacin plays an important role in the regulation of autophagic flux and protects tumor cells against TRAIL-mediated apoptosis. Our results indicated that niacin activated autophagic flux in human colon cancer cells and the autophagic flux activation protected tumor cells from TRAIL-induced dysfunction of mitochondrial membrane potential and tumor cell death. We also demonstrated that ATG5 siRNA and autophagy inhibitor blocked the niacin-mediated inhibition of TRAIL-induced apoptosis. Taken together, our study is the first report demonstrating that niacin inhibits TRAIL-induced apoptosis through activation of autophagic flux in human colon cancer cells. And our results also suggest that autophagy inhibitors including genetic and pharmacological tools may be a successful therapeutics during anticancer therapy using TRAIL.

## INTRODUCTION

Niacin, also known as vitamin B3, niacinamide and nicotinamide, is a water-soluble vitamin that humans are unable to synthesize in sufficient amounts [1]. Nicotinamide can block drug-induced apoptosis in human cortical neuronal cells [2]. An absence of niacin causes the deficiency disease pellagra, dementia, diarrhea, and skin problems such as dermatitis [3]. Treatment with niacin has the potential to impair genomic stability and enhances the risk for cancer by regulation of intracellular calcium signaling pathways [4]. Niacin enhances the NAD^+^/NADH ratio and induces therapeutic normalization of NAD^+^/NADH balance through autophagy in human breast adenocarcinoma cells [5]. Moreover, treatment with niacin activates the PI3K/Akt cascade in the A431 human epithelial carcinoma cell line [6].

Apoptosis is a form of programmed cell death that occurs in multicellular organisms and is associated with changes in cell morphology such as blebbing, cell shrinkage, nuclear fragmentation, chromatin condensation, and chromosomal DNA fragmentation [7, 8]. Defective apoptotic signaling has been implicated in a variety of diseases [9, 10]. Apoptosis-related factors such as fas and caspases enhance apoptosis, whereas several members of the Bcl-2 family inhibit apoptosis [11, 12].

Tumor necrosis factor-related apoptosis-inducing ligand (TRAIL), a member of the TNF super family, is a potential cancer therapeutic agent that can induce apoptosis [13–15]. Recent studies found that binding of TRAIL to death receptor 4 (DR4) and DR5 induces DR clustering, and subsequent recruitment of FADD and activation of caspase-8 induces apoptosis [16]. TRAIL also binds to decoy receptors 1 and 2, which inhibits the induction of apoptosis [17]. Cell surface death receptors, including DR4 and DR5, induce apoptosis through DISC, which recruits the protease caspase-8 [18]. Increased death receptor expression is associated with survival in patients with colon cancer [19, 20]. TRAIL has been shown to induce apoptotic cell death in various cancer cells by binding to DR4 and DR5 to activate the extrinsic apoptosis pathway [21]; in contrast, most normal cells are relatively resistant to TRAIL treatment [22, 23].

Treatment with TRAIL has been shown to induce autophagy-dependent cell death in TRAIL-resistant cancer cells [24, 25]. Beclin 1, a known key factor in autophagy, has important roles in the crosstalk with anti-apoptotic proteins such as BCL-2 [26]. Autophagy is a lysosomal degradation process that can mediate cell death and cell survival [27]. Autophagy prevents cell death during hypoxia, starvation, growth factor deprivation, endoplasmic reticulum (ER) stress, and microbial infection [28]. However, autophagy can also be involved in cell death resulting from caspase activation, lysosomal membrane permeabilization, and dysregulation of the mitochondrial membrane potential [29]. Autophagy occurs in all nucleated type cells and the process of autophagic flux is essential in animal, plant, and yeast cells [30–32]. Activation of autophagy has been shown to prevent cell death in PCa cells whereas inhibition of autophagy enhances reagent-induced cell death [33, 34]. Autophagic cell death induced by neurodegenerative disease is characterized by the accumulation of autophagic organelles such as autophagosomes and autophagolysosomes [35–37]. Basically, autophagy plays an important role in cellular homeostasis through the degradation of long-lived proteins, intracellular organelles, and misfolded proteins but excessive autophagy can induce cellular destruction [35, 36]. Atg12-Atg5-Atg16 complex is associated with formation of the autophagosome and Light chain 3 (LC3, also known as ATG8) is an autophagy marker that is lapidated during induction of autophagic flux and is required for autophagosome formation [38, 39]. Another autophagy marker, p62/SQSTM1, plays an important role in the degradation of polyubiquitinated substrates by autophagy flux, thus causing its own degradation [40]. Recently, TRAIL-resistant cells were shown to exhibit high autophagic flux with increased clearance of p62 protein; however, TRAIL-sensitive cells display low autophagic flux and accumulation of p62 [41]. Furthermore, p62/SQSTM1 binds to DISC and enhances the activation, aggregation, and processing of caspase-8, a known pro-apoptotic factor [18].

Here, we show that niacin treatment decreased the expression of death receptors 4 and 5 and induced autophagy flux in human colon cancer cells. In turn, inhibition of autophagy flux by chloroquine sensitized cells to TRAIL-induced apoptosis and increased expression of DR4 and DR5 proteins with niacin treatment. These results identify a mechanism by which activation of autophagy flux induced by niacin treatment inhibits the anticancer activity of TRAIL in human colon cancer cells.

## RESULTS

### Niacin inhibits TRAIL-induced apoptosis in HCT116 cells

**Figure 1:**
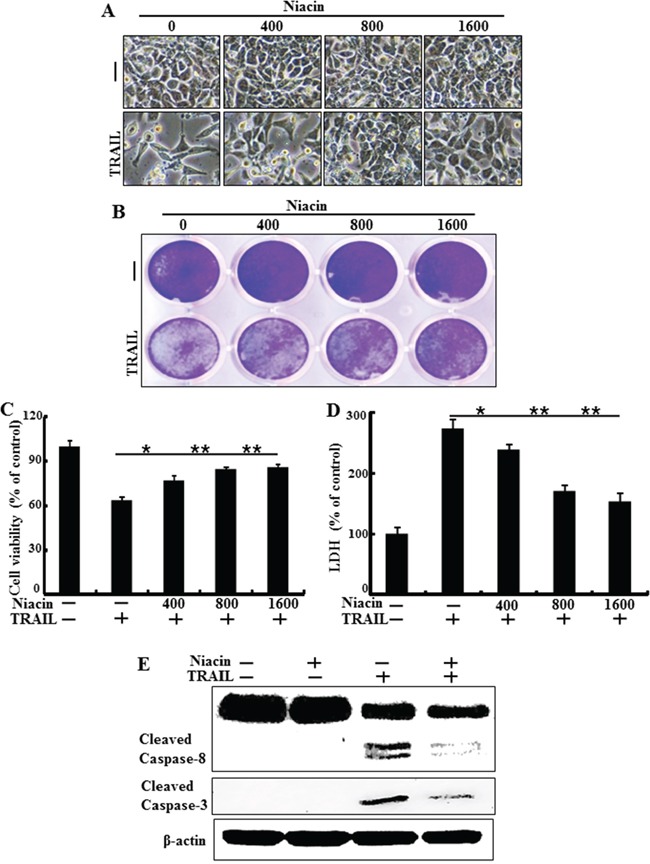
Niacin inhibits TRAIL-induced apoptosis in HCT116 cells HCT116 cells were pretreated with niacin (400–1,600 μM) for 12 h and then incubated with recombinant TRAIL (100 ng/ml) for an additional 2 h. **A.** Cells were photographed under light microscopy (×200). **B, C.** Viable cells were stained with crystal violet. Viability of control cells was set at 100%, and viability relative to the control was estimated. Results are representative of three independent experiments. **D.** Release of LDH into the cell culture supernatant. **E.** HCT116 cells were pretreated with niacin for 12 h, and then further co-incubated with or without recombinant TRAIL protein (100 ng/ml) for an additional 2 h. Whole cell lysates were subjected to western blot analysis of caspase-8 and caspase-3. β-actin was used as a loading control. *p < 0.05, **p < 0.001; significant differences between control and each treatment group. adj. volume, adjustment of volume (band volume minus background volume).

To understand the effect of niacin treatment on TRAIL-induced apoptosis in HCT116 human colon cancer cells, we examined changes in cell morphology and viability using light microscopy, crystal violet assay, and LDH release assay. The cells were pretreated with the indicated doses of niacin for 12 h and treated with TRAIL for an additional 2 h. TRAIL treatment alone significantly increased cell death, indicating that HCT116 cells are sensitive to TRAIL treatment. However, niacin inhibited TRAIL-induced cell death in a dose-dependent manner. The both cell morphology data and crystal violet showed that the combination of TRAIL with niacin decreased the number of apoptotic cells compared with TRAIL alone (Figure 1A and Figure 1B). The niacin and TRAIL co-treatment increased cell viability and decreased LDH release (Figure 1C and Figure 1D). As shown in Figure 1E, niacin and TRAIL co-treatment also decreased production of the activated forms of apoptotic factors, including caspase-8 and caspase-3, compared to treatment with TRAIL alone (Figure 1E). Overall, these data demonstrated that niacin treatment inhibits TRAIL-mediated apoptosis in TRAIL-sensitive HCT116 cells.

### Niacin decreases expression of death receptor proteins

**Figure 2:**
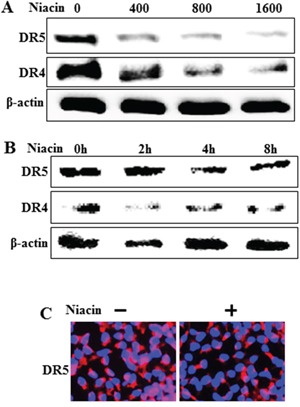
Niacin decreased expression of death receptor protein **A, B.** HCT116 cells were treated with niacin at 400–1,600 μg/ml for 12 h and subjected to western blot analysis of DR4 and DR5 proteins. β-actin was used as a loading control. **C.** Representative images of DR5 protein expression in HCT116 cells.

TRAIL interacts with specific receptors known as death receptor 4 (TRAIL-R1 or DR4) and death receptor 5 (TRAIL-R2 or DR5) [17, 44]. To investigate the effect of niacin on TRAIL-related expression of death receptor proteins we treated HCT116 cells with niacin in a dose-dependent manner for 12 h and a time-dependent manner for 2–8 h. Cell lysates were subjected to western blot analysis to determine changes in DR4 and DR5 protein expression (Figure 2A and Figure 2B). Western blot analysis and immunofluorescence staining revealed that niacin treatment decreased expression of DR4 and DR5 protein compared to control (Figure 2B and 2C).

### Niacin induced activation of autophagic flux

**Figure 3:**
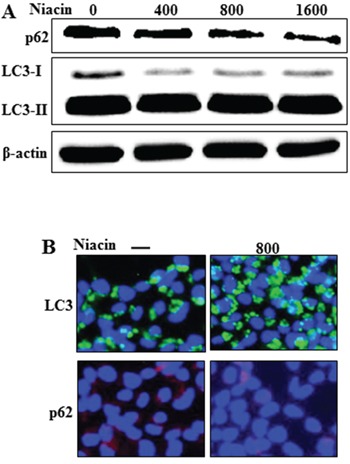
Niacin induced autophagic flux **A.** HCT116 cells were treated with niacin at 400–1,600 μg/ml for 12 h and subjected to western blot analysis of p62, LC3-I, and LC3-II proteins. β-actin was used as a loading control. **B.** Representative images of p62 and LC3 protein expression in HCT116 cells.

Recent studies found that autophagic flux is involved in the activation of apoptotic signaling factors such as cleaved caspase-3 and cleaved caspase-8 in TRAIL-mediated apoptosis [18, 45, 46]. Therefore, we evaluated expression of autophagic flux markers including LC3 and p62 proteins by western blot analysis and immunofluorescence staining (Figure 3). Western blot analysis showed that the expression of p62 protein decreased and that of LC3-II protein increased after niacin treatment in a dose-dependent manner (Figure 3A). During the autophagy process, microtubule-associated light chain 3 (LC3-I) is converted into the autophagosomal membrane form of LC3-II, which is the most reliable marker for autophagy activation [47]. p62 protein facilitates the degradation of polyubiquitinated protein or organelles, causing its own degradation; therefore, a decreased level of p62 protein indicates activation of autophagy and autophagic degradation [40]. Our western blot data demonstrated that niacin treatment induced autophagic flux in HCT116 human colon cancer cells. Immunofluorescence staining confirmed that niacin treatment decreased accumulation of p62 protein (Figure 3B). Collectively, these results demonstrated that niacin induced autophagic flux in human colon cancer cells, which rendered the cells resistant to TRAIL-mediated apoptosis.

### Inhibition of autophagic flux induced by chloroquine blocks the protective function of niacin

**Figure 4:**
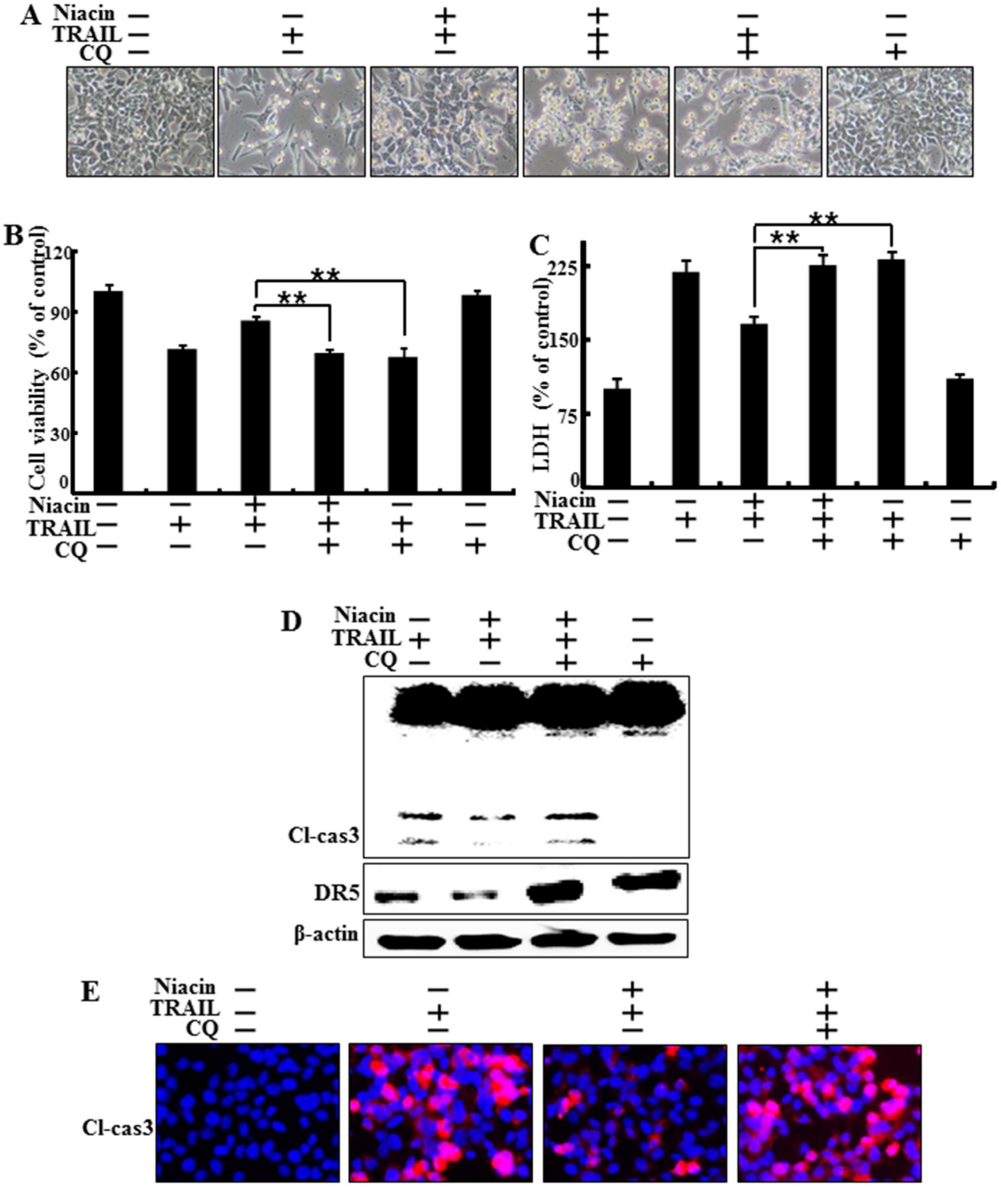
Inhibition of autophagy blocked the protective function of niacin treatment HCT116 cells were pretreated with 50 nM chloroquine for 6 h and then exposed to 800 μM niacin for 12 h and 100 ng/ml TRAIL for 2 h. **A.** Cell morphology was photographed under light microscopy (×200). **B.** Viability of treated cells was measured by crystal violet staining. Viability of control cells was taken as 100%. **C.** LDH release into the cell culture medium was measured after exposure to TRAIL for 3 h. **D.** Western blot analysis of caspase-3. β-actin was used as a loading control. **E.** Representative images of cleaved caspase-3 expression in HCT116 cells. Bar graph indicates the total number of cells and percentage of apoptotic cells. *p < 0.05 or **p < 0.01 indicate significant differences between control and each treatment group.

Next, we investigated the effect of combined treatment with niacin and chloroquine, a known autophagy inhibitor, on TRAIL treatment. HCT116 cells were pretreated with 50 nM chloroquine for 6 h and exposed to 800 μM niacin for 12 h. Cells were then treated with 100 ng/ml TRAIL protein for 2 h. We examined cell morphology, cell viability, and LDH release using light microscopy and crystal violet assay. Pharmacological inhibition of autophagy by chloroquine in the presence of niacin sensitized HCT116 cells to TRAIL-induced cell death compared to niacin alone (Figure 4A–4C). And also, chloroquine increased TRAIL induced apoptosis and chloroquine alone was not affected cell viability (Figure 4A–4C). The activated form of caspase-3, which is known to be a pro-apoptotic factor, was increased by chloroquine in co-treatment with niacin and TRAIL. Furthermore, death-receptor5 protein increased by chloroquine treatment (Figure 4D). Immunofluorescence staining confirmed that chloroquine treatment increased production of the active form of caspase-3 (Figure 4E). These data indicated that inhibition of autophagy by chloroquine increased TRAIL-related proapoptotic signaling in HCT116 cells.

### Chloroquine inhibits autophagic flux with or without TRAIL treatment

**Figure 5:**
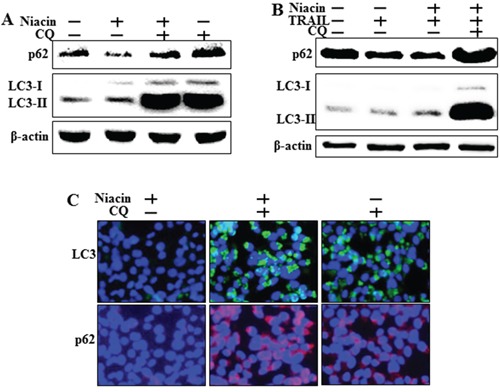
Chloroquine inhibits autophagic flux with or without TRAIL treatment HCT116 cells were pretreated with 50 nM chloroquine for 6 h and then exposed to 800 μM niacin for 12 h and 100 ng/ml TRAIL for 2 h. **A, B.** Western blot analysis of p62 and LC3. β-actin was used as a loading control. **C.** Representative images of LC3 and p62 protein expression in HCT116 cells.

Next, we confirmed inhibition of autophagic flux by measuring protein expression of the autophagy markers p62 and LC3 by western blot analysis and immunofluorescence staining. Western blot analysis showed that chloroquine increased levels of both p62 and LC3-II with or without TRAIL treatment (Figure 5A–5B). Immunofluorescence staining confirmed that chloroquine increased p62 and LC3-II levels compared to treatment with niacin alone (Figure 5C). These data indicated that chloroquine treatment inhibited the niacin-induced degradation of p62 and LC3-II protein.

### Genetic inhibition of autophagy promotes TRAIL-induced cell death upon niacin treatment

**Figure 6:**
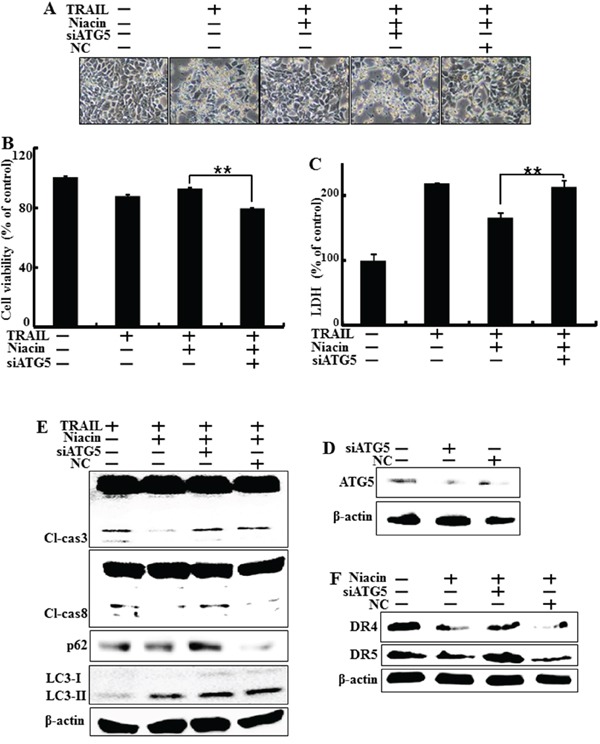
Genetic inhibition of autophagy promotes TRAIL-induced cell death upon niacin treatment HCT116 cells were pre-treated with 20 nM ATG5 siRNA for 4 h and then exposed to 800 μM niacin for 12 h and treated with 100 ng/ml TRAIL for 2 h. **A.** Cell morphology was photographed under light microscopy (×200). **B.** Viability of treated cells was measured by crystal violet staining. Viability of control cells was taken as 100%. **C.** LDH release into the cell culture medium was measured after exposure to TRAIL for 2 h. **D.** Western blot analysis of ATG5 protein confirmed specific protein knockdown. β-actin was used as a loading control. **E.** Western blot analysis of caspase-3 and caspase-8. β-actin was used as a loading control. **F.** Western blot analysis data showed DR4 and DR5 protein known as TRAIL related death receptor. β-actin was used as a loading control. *p < 0.05 or **p < 0.01 significant differences between control and each treatment group.

To verify that niacin-induced autophagy plays a protective role against TRAIL-induced apoptosis, we used a genetic approach (ATG5 siRNA) to study the effect of autophagy inhibition on TRAIL-induced apoptosis in HCT116 cells. Atg5 is necessary for autophagy because it's role in autophagosome elongation [47]. The cells were pretreated with 20 nM ATG5 siRNA for 24 h and then treated with 800 μM niacin for 12 h with or without treatment with 100 μg/ml TRAIL for 2 h. Examination of cell morphology showed that ATG5 siRNA inhibited the effect of niacin treatment on TRAIL-induced apoptosis (Figure 6A). We further studied the sensitivity of HCT116 cells to TRAIL-induced cell death using crystal violet and LDH assays (Figure 6B–6C), and showed that ATG5 siRNA down-regulated cell viability and increased LDH levels (Figure 6B–6C). Functional knockdown of siRNA was confirmed by western blot data showing down-regulation of ATG5 protein by ATG5 siRNA (Figure 6D). Next, we investigated whether ATG5 siRNA affects the expression of proapoptotic factors such as caspase-8 and caspase-3 protein using western blot analysis. Expression of ATG siRNA in HCT116 cells increased the induction of cleaved caspase-8 and cleaved caspase-3 upon treatment with niacin and TRAIL, and increased the expression of autophagy markers including p62 and LC3 (Figure 6E). Furthermore, ATG5 siRNA increased death receptor4 and 5 compared to niacin treatment (Figure 6F). These results showed that genetic inhibition of autophagy by ATG5 siRNA enhanced TRAIL-induced apoptosis and inhibited autophagic flux in HCT116 cells.

### Effect of niacin on mitochondria membrane potential and p-Akt

**Figure 7:**
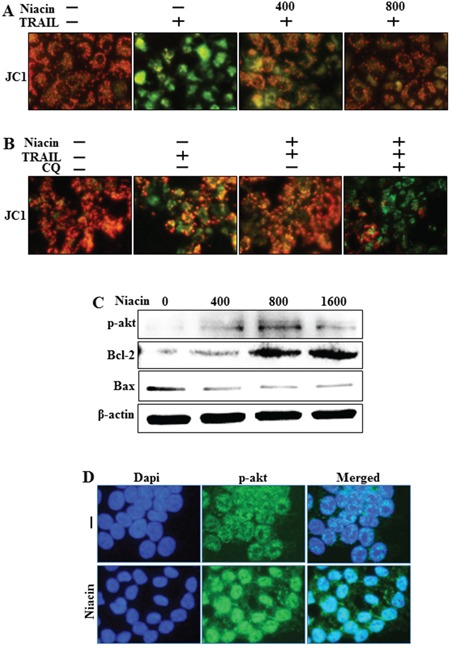
Effect of niacin on mitochondria membrane potential HCT116 cells were exposed to niacin (400–1,600 μM) for 12 h. **A and B.** Representative images of JC-1 aggregate formation in the treated cells. JC-1 aggregate forms (red) and mono forms (green) were measured in the treated cells by confocal microscopy analysis; scale bar, 50 μm. **C.** Western blot analysis of phospho-Akt, a known survival signaling protein, and the proapoptotic markers BCL-2 and Bax. β-actin was used as a loading control. **D.** Representative images of phospho-Akt protein expression in HCT116 cells.

Recent studies showed that niacin affects mitochondrial complex 1 activity and enhances the NAD^+^/NADH ratio [5]. Furthermore, niacin treatment promotes activation of the PI3/Akt cascade in human epithelial carcinoma cells [6]. Therefore, we examined the mitochondrial transmembrane potential and expression of p-Akt and mitochondria-related apoptotic factors including BCL-2 and Bax. Cells were pretreated with the indicated doses of niacin for 12 h and protein levels of BCL-2, Bax, and p-Akt were examined by western blot analysis. We examined mitochondrial dysfunction using JC-1 and the data exhibited that niacin decreased TRAIL-induced mitochondrial dysfunction but chloroquine increased mitochondrial dysfunction upon niacin treatment (Figure 7A–7B). Next, Our western blot data and immunofluorescence staining data showed that niacin treatment significantly increased the protein expression of p-Akt, which is known to be a survival signaling factor in cancer cells [48] (Figure 7C–7D). The intrinsic apoptotic pathway is characterized by mitochondrial permeabilization and involves activation of proapoptotic proteins including BCL-2 and Bax [49]. Western blot analysis data showed that niacin increased levels of BCL-2 and decreased levels of Bax (Figure 7C). These data indicated that niacin has a potential role in protecting against intrinsic apoptosis signaling and increasing survival.

## DISCUSSION

The goal of this study was to investigate the effect of niacin on TRAIL treatment in TRAIL-sensitive cells. Our study suggests that niacin induces autophagic flux in TRAIL-induced apoptosis through down-regulation of death receptors proteins DR4 and DR5 and prevention of mitochondria membrane depolarization.

Some studies have suggested that niacin treatment activates AKT protein, a cell survival factor in human epithelial carcinoma cell lines, and inhibits metastatic activity by enhancing the NAD^+^/NADH ratio in cultured MDA-MB-435 and MDA-MB-231 human breast carcinoma cells [5, 6]. As a potential therapeutic drug, TRAIL induces apoptotic programed cell death in various cancer cells, and HCT116 colon cancer cells are known to be sensitive to TRAIL [50].

However, until now the relationship between niacin and TRAIL has not been studied in any cancer cells. Our results showed that TRAIL treatment induced apoptosis in HCT116 cells, and that niacin treatment inhibited TRAIL-induced apoptosis and decreased expression of the proapoptotic factors caspase-8 and caspase-3 (Figure 1). Niacin is known to affect the activation of mitochondrial complex 1 and increase the NAD^+^/NADH ratio in breast cancer cells; therefore, we examined the effect of niacin on mitochondria membrane potential in HCT cells. Niacin treatment increased the expression of Bcl-2 protein, a known anti-apoptotic factor, and decreased the expression of decreased Bax, a known proapoptotic factor (Figure 7A). Niacin also inhibited the induction by TRAIL of the JC-1 monomer forms, a marker of cell apoptosis (Figure 7D). Furthermore, niacin down-regulated death receptor proteins including DR4 and DR5 that are related to TRAIL in the HCT116 TRAIL-sensitive cell line (Figure 3).

Recent studies have shown that TRAIL induces autophagic flux in TRAIL-resistant cells and inhibits autophagic flux in TRAIL-sensitive cells [41]. Niacin decreases expression of the autophagy marker p62 in breast cancer cells [5] but the relationship between niacin and autophagy is unclear in a variety of cancer cells, including HCT116 TRAIL-sensitive cells. We observed that niacin markedly decreased the level of p62 protein and increased that of LC3-II, indicating autophagy induction (Figure 3). To confirm that niacin inhibited TRAIL-mediated cell death via autophagic flux, we showed that pharmacological or genetic inhibition of autophagy promoted TRAIL-induced cell death compared to niacin alone using chloroquine as a lysosomal inhibitor and siRNA specific for the autophagosomal factor ATG5 (Figure 5 and Figure 6). Moreover, combined niacin and chloroquine treatment inhibited JC-1 aggregation as a survival marker and induced the JC-1 monomer form as an apoptosis marker (Figure 7B).

Niacin (also known as vitamin B3 or nicotinic acid) is known to be present in black beans and other common foods, and has been shown inhibit metastasis of breast cancer cells by modulation of NAD^+^ levels [5, 51]. However, the relationship between niacin and apoptosis induced by anticancer reagents such as TRAIL protein has not been studied in cancer cells. We found that niacin treatment inhibited TRAIL-mediated apoptosis and induced autophagy flux in HCT116 human colon cancer cells. We examined that both niacin treatment and niacin combined with chloroquine were not affected death receptors of mRNA levels (data not shown). These result indicated that autophagy induced by niacin treatment influence on death receptor of protein levels. Furthermore, autophagic pathway is associated with lysosomal degradation of protein. Therefore, niacin mediated autophagic flux effects death receptors of protein expression. Inhibition of autophagic flux by chloroquine and ATG5 siRNA decreased the protective effect of niacin. These results demonstrate that niacin inhibits TRAIL-induced cell death through regulation of autophagic flux in TRAIL-sensitive HCT116 cells, suggesting that niacin treatment might have adverse effects on anticancer agents that function through the induction of autophagy flux. So, TRAIL treatment has to combine with autophagy inhibitor such as chloroquine in human cancer therapy.

## MATERIALS AND METHODS

### Cell culture

The human colon carcinoma cell line HCT116 was maintained in RPMI1640 medium containing 10% fetal bovine serum (FBS; Invitrogen-Gibco, Carlsbad, CA, USA) and 100 μg/ml penicillin-streptomycin in a humidified incubator maintained at 37°C and 5% CO_2_.

### Protein isolation and western blotting

Proteins were resolved by 10–15% sodium dodecyl sulfate-polyacrylamide gel electrophoresis, transferred to nitrocellulose membranes, and analyzed by western blotting as described previously [42]. The antibodies used for immunoblotting were specific for Bcl-2 (Santa Cruz Biotechnology, Santa Cruz, CA, USA); phosphorylated-Akt (p-Akt), cleaved caspase-3, caspase-8, p62 and LC3(Cell Signaling Technology, Danvers, MA, USA); DR4, DR5 and β-actin (Sigma-Aldrich, St Louis, MO, USA).

### Crystal violet assay

Whole cells were plated at 1 × 10^4^ cells/well in a 12-well plate and incubated at 37°C for 24 h. The cells were pretreated with chloroquine for 6 h and niacin for 12 h and further incubated with recombinant TRAIL for an additional 2 h. Cell morphology was examined using an inverted microscope (Nikon, Japan) and cell viability was determined by the crystal violet staining method as previously described [43]. Briefly, cells were stained for 10 min at room temperature with a staining solution (0.5% crystal violet in 30% ethanol and 3% formaldehyde), washed four times with water, and dried. Cells were then lysed with 1% SDS solution and absorbance was measured at 550 nm. Cell viability was calculated from the relative dye intensity and compared to the controls.

### Lactate dehydrogenase assay

Cytotoxicity was assessed by performing an LDH assay on cell supernatant using a LDH Cytotoxicity Detection kit (Takara Bio, Tokyo, Japan) according to the manufacturer's protocol. LDH activity was determined by measuring absorbance at 490 nm.

### Immunofluorescence staining

Cell lines cultured on glass coverslips were treated with niacin and chloroquine under normoxia. The cells were washed with PBS and fixed with cold acetone for 90 sec at room temperature. The cells were then washed with PBS again, blocked with 5% fetal bovine serum in Tris-buffered saline with Tween, and incubated with monoclonal antibodies against p62, LC3, DR5 and cleaved caspase-3 (2 μg/ml) for 24 h at room temperature. Unbound antibody was removed by an additional wash with PBS, and the cells were incubated with labeled anti-mouse Alexa Fluor 546 (for anti-p62) IgG antibody (4 μg/ml), and anti-rabbit Alexa Flour 488 (for anti-LC3, DR5 and cleaved-caspase3) for 2 h at room temperature. Finally, the cells were mounted with DakoCytomation medium and visualized by fluorescence microscopy.

### RNA interference

HCT116 cells were transfected with ATG5-specific small interfering RNA (siRNA; Stealth RNAi, Santa Cruz Biotechnology) using Lipofectamine 2000 according to the manufacturer's instructions. The cells were plated in 24-well plates, pretreated with 20 nM ATG5 siRNA for 24 h, and incubated with recombinant TRAIL (0–100 ng/ml) for an additional 3 h under the same conditions. Scrambled ATG5 siRNA (Invitrogen) was used as the negative control.

### Mitochondrial transmembrane potential (MTP) assay

The changes in MTP were evaluated using a cationic fluorescent indicator (JC-1; Molecular Probes, Eugene, OR, USA), which aggregates in intact mitochondria (red fluorescence) indicating high or normal MTP and low MTP when it remains in a monomeric form in the cytoplasm (green fluorescence). HCT116 human colon cancer cells were incubated in MEM containing 10 ml JC-1 at 37°C for 15 min, washed with PBS and subsequently transferred to a clear 96-well plate. JC-1 aggregate fluorescence emission was measured at 583 nm, with an excitation wavelength of 526 nm. JC-1 monomer fluorescence intensity was also measured with both excitation and emission wavelengths at 525 and 530 nm, respectively using a microplate reader (SpectraMax M2; Molecular Devices) or a Guava easyCyte HT System. HCT116 human colon cancer cells were cultured on cover slips in a 24-well plate, incubated in MEM containing 10 ml JC-1 at 37°C for 15 min and then washed with PBS. Finally, the cells were mounted with DakoCytomation fluorescent mounting medium and visualized under a fluorescence microscope.

### Statistics

All data are expressed as means ± standard deviation (SD). Comparisons were performed using the Student's *t*-test and the ANOVA Duncan test with the SAS statistical package (SAS Institute, Cary, NC, USA). The results were considered significant at *P* < 0.05 (*) or *P* < 0.01 (**).
